# Olaparib Induces RPL5/RPL11-Dependent p53 Activation *via* Nucleolar Stress

**DOI:** 10.3389/fonc.2022.821366

**Published:** 2022-06-03

**Authors:** Tao Han, Jing Tong, Mengxin Wang, Yu Gan, Bo Gao, Jiaxiang Chen, Youxun Liu, Qian Hao, Xiang Zhou

**Affiliations:** ^1^ School of Basic Medical Sciences, Xinxiang Medical University, Xinxiang, China; ^2^ Fudan University Shanghai Cancer Center, Fudan University, Shanghai, China; ^3^ Department of Oncology, Shanghai Medical College, Fudan University, Shanghai, China; ^4^ Department of Physiology, Medical College of Nanchang University, Nanchang, China; ^5^ Key Laboratory of Breast Cancer in Shanghai, Fudan University Shanghai Cancer Center, Fudan University, Shanghai, China; ^6^ Shanghai Key Laboratory of Medical Epigenetics, International Co-laboratory of Medical Epigenetics and Metabolism (Ministry of Science and Technology), Institutes of Biomedical Sciences, Fudan University, Shanghai, China

**Keywords:** p53, ribosomal protein (RP), nucleolar (ribosomal) stress, MDM2, PARP inhibitior

## Abstract

The poly (ADP-ribose) polymerase (PARP) inhibitor (PARPi) Olaparib is a widely used targeted therapy for a variety of solid tumors with homologous recombination deficiency (HRD) caused by mutation of *BRCA1/2* or other DNA repair genes. The anti-tumor activity of Olaparib has been largely attributed to its ability to inhibit PARP enzymes and block DNA single-strand break (SSB) repair, which eventually leads to the most detrimental DNA damage, double-strand breaks (DSB), in HRD cells. Although PARPi was found to induce p53-dependent cell death, the underlying molecular mechanism remains incompletely understood. Here, we report that Olaparib treatment leads to p53 stabilization and activation of its downstream target genes in a dose- and time-dependent manner. Mechanistically, Olaparib triggers nucleolar stress by inhibiting biosynthesis of the precursor of ribosomal RNAs (pre-rRNA), resulting in enhanced interaction between ribosomal proteins (RPs), RPL5 and RPL11, and MDM2. Consistently, knockdown of RPL5 and RPL11 prevents Olaparib-induced p53 activation. More importantly, Olaparib efficiently suppresses breast and colorectal cancer cell survival and proliferation through activation of p53. Altogether, our study demonstrates that Olaparib activates the nucleolar stress-RPs-p53 pathway, suggesting rRNA biogenesis as a novel target for PARPi.

## Introduction

Mutation of DNA damage repair (DDR) genes is closely associated with predisposition of different types of cancer (1, 2). Both *BRCA1* and *BRCA2* are crucial to homologous recombination (HR) that is widely used by cells to repair the most detrimental DNA damage, DNA double-strand breaks (DSB). Germline mutations in *BRCA1/2* are highly prevalence in breast cancer, ovarian cancer and many other types of cancer, including lymphoma, leukemia, melanoma, prostate, pancreatic, stomach, and colorectal cancer (3–6). Poly(ADP-ribose) polymerase-1 (PARP-1) is a ubiquitous nuclear enzyme involved in multiple biological processes, such as DNA repair, cell cycle, and apoptosis (7). It was found that the expression of PARP is significantly upregulated in various cancer cell lines and tumor tissues from patients (8–10). Recently, growing evidence has demonstrated that inhibition of PARP is a promising targeted therapy for cancer patients with deficiency in *BRCA1/2* or other DDR genes (11, 12). The first PARP inhibitor (PARPi) Olaparib has been successively used for treatment of patients with advanced solid tumors carrying a germline *BRCA1/2* mutation (13, 14). It has been shown that Olaparib achieves its therapeutic efficacy *via* several mechanisms. PARPi impairs PARP activity to mediate protein PARylation that facilitates recruitment of DNA repair components to the single-strand break (SSB) sites (7, 9). Also, PARPi was found to impede DNA replication by destabilizing replication forks, resulting in replication stress and subsequent cell death (12, 15). Furthermore, PARPi is able to induce PARP trapping, a process involving formation of a stable complex of PARPi and PARP at SSB lesions, leading to disruption of the recycle of PARP in the DDR cascade (16, 17). PARPi is believed to prevent SSB damage that may turn into DSB through aberrant DNA replication. Thus, tumor cells with HRD are particularly vulnerable to PARPi based on the genetic concept of synthetic lethality.

The tumor suppressor p53 plays an important role in DNA damage response. As a transcription factor, p53 activates the expression of a wealth of genes involved in cell cycle arrest, DNA repair, and apoptosis (18, 19). As excessive p53 activity is extreme cytotoxic, surveillance mechanisms are employed by cancer cells to inactivate p53. For instance, the E3 ubiquitin ligase MDM2 maintains a proper low level of p53 by promoting its ubiquitination and proteasomal degradation (20–24). In addition, mutations of the *TP53* gene occur in around 50% of human cancers, which not only abrogates tumor suppressive activity of p53, but also renders “gain-of-function” to drive cancer development (25). Recently, several studies have indicated that p53 activity may enhance tumor response to PARPi, as these agents can activate p53 to trigger apoptosis and ferroptosis (26, 27) or repress RAD51-mediated HR repair (28). Additionally, we have recently demonstrated that long noncoding RNA RMRP is an inhibitor of p53 in response to PARPi treatment, while targeting RMRP significantly bolsters p53 activation and enhances tumor sensitivity to PARPi (29, 30). However, the mechanisms underlying how PARPi induces p53 activation are still incompletely understood.

In this study, we reveal that Olaparib treatment induces p53 stabilization and activation in time- and dose-dependent manner. Interestingly, Olaparib represses ribosomal RNA (rRNA) biosynthesis, consequently leading to nucleolar stress (or ribosomal stress). It has been well-documented that perturbation of ribosome biogenesis promotes translocation of ribosomal proteins (RPs) from the nucleolus to the nucleus where they can associate with MDM2 and inhibit MDM2-mediated p53 degradation (31–34). Herein, we elaborate that Olaparib treatment enhances interaction between RPL5/RPL11 and MDM2, whereas knockdown of RPL5 or RPL11 impairs Olaparib-induced p53 activation. Consistently, HCT116 ^p53+/+^ cells exhibit higher sensitivity to Olaparib than HCT116 ^p53−/−^ cells. Therefore, our study unveils rRNA biogenesis as an alternative target of PARPi, and demonstrates a novel action mode of PARPi *via* the nucleolar stress-RPs-p53 axis.

## Materials and Methods

### Cell Culture and Olaparib Treatment

Human Colorectal Cancer cell lines HCT116^p53+/+^, HCT116^p53-/-^ and Breast cancer cell line Cal51 were cultured in Dulbecco’s modified Eagle’s medium supplemented with 10% fetal bovine serum, 50 units/ml penicillin, and 0.1 mg/ml streptomycin and maintained at 37°C in a 5% CO_2_ humidified atmosphere. The cells were treated with different doses of Olaparib (MCE, Shanghai, China) and harvested at indicated time courses or dose for the future experiments.

### SiRNAs and Antibodies

The siRNA sequences were used in this paper as below, siNC: UUCUCCGAACGUGUCACGU, siRPL5: 5’-GGAGGAGAUGUAUAAGAAATT-3’, siRPL11: 5’-GGAACUUCGCAUCCGCAAATT-3’. All siRNAs were synthesized by Genepharma company (Shanghai, China). The anti-p53 (Catalog sc-126, Santa Cruz Biotechnology), anti-MDM2 (Catalog# M4308, Sigma), anti-p21 (Catalog#2947, Cell Signaling Technology), anti-RPL5 (Catalog ab86863, Abcam), anti-RPL11 (Catalog ab79352, Abcam), anti-GAPDH (Catalog 60004-1-Ig, Proteintech), anti-β-actin (Catalog ARG62346, Proteintech), anti-α-tubulin (Catalog 66031-1-Ig, Proteintech) were commercially purchased.

### Immunoblot and Co-Immunoprecipitation Assays

Cells were lysed with lysis buffer consisting of 50 mM Tris/HCl (pH7.5), 0.5% Nonidet P-40 (NP-40), 1 mM EDTA, 150 mM NaCl, 1 mM dithiothreitol (DTT), 0.2 mM phenylmethylsulfonyl fluoride (PMSF),10 μM pepstatin A and 1 μg/ml leupeptin. Equal amounts 60 μg of clear cell lysates were used for immunoblot analysis. Co-IP assays were conducted using antibodies as indicated in the figure legends. In brief, 1 mg of total proteins were incubated with the indicated antibody at 4°C for overnight, and then Protein A or G beads were added and the mixture was left to incubate at 4°C for additional 2 h. At last, the beads were washed five times with lysis buffer. Bound proteins were detected by IB with antibodies as indicated in the figure legends.

### Immunofluorescence Assay

Cells were fixed with methanol in −20°C for overnight. The fixed cells were washed by PBS and blocked with 8% BSA in PBS for 1 h followed by incubation with the anti-Flag antibody in 2% BSA in 4°C for overnight. The cells were then washed and incubated with the secondary antibody and DAPI.

### Reverse Transcription and Quantitative RT-PCR Analyses

Total RNA was isolated from cells using RNAiso Plus (Takara, Dalian, Liaoning, China) according to the manufacturer’s protocol. Total RNAs of 1μg were used as templates for reverse transcription reaction using PrimeScript RT reagent Kit with gDNA Eraser (Takara, Dalian, Liaoning, China). Quantitative RT-PCR was conducted using ChamQ SYBR qPCR Master Mix (Novazyme, Nanjing, China) according to the manufacturer’s protocol. The following primers were used as below: Actin-F: 5’-CATGTACGTTGCTATCCAGGC-3’, Actin-R: 5’-CTCCTTAATGTCACGCACGAT-3’, Puma-F: 5’-GACCTCAACGCACAGTACGAG-3’, Puma-R: 5’-AGGAGTCCCATGATGAGATTGT-3’, BTG2-F: 5’-ACGGGAAGGGAACCGACAT-3’, BTG2-R: 5’-CAGTGGTGTTTGTAGTGCTCTG-3’, MDM2-F: 5’-GAATCATCGGACTCAGGTACATC-3’, MDM2-R: 5’-TCTGTCTCACTAATTGCTCTCCT-3’, BAX-F: 5’-CCCGAGAGGTCTTTTTCCGAG-3’, BAX-R: 5’-CCAGCCCATGATGGTTCTGAT-3’, p21-F: 5’-CTGGACTGTTTTCTCTCGGCTC-3’, p21-R: 5’-TGTATATTCAGCATTGTGGGAGGA-3’, 112-bp-F: 5’-TGAGAAGACGGTCGAACTTG-3’, 112-bp-R: 5’-TCCGGGCTCCGTTAATGATC-3’, 96-bp-F: 5’-GGCCATACCACCCTGAACGC-3’, 96-bp-R: 5’-CAGCACCCGTATTCCCAGG-3’. The 112-bp pre-rRNA fragment encompasses 5’-external transcribed sequence (5’-ETS) and 18S rRNA. The 96-bp pre-rRNA fragment is from 18S rRNA to internal transcribed sequence-1 (ITS-1) (35). The primers were sythesized by GENEWIZ (Suzhou, China).

### RNA Interference

RNA interference-mediated knockdown of endogenous RPL5 and RPL11 were performed as described previously (33). These siRNA duplexes were introduced into cells using Hieff Trans liposomal transfection reagent (Yeasen, Shanghai, China) according to the manufacturer’s protocol. The transfected cells were treated with or without 10 μM of Olaparib for 24 h before harvesting. Cells were harvested at 48 h of post transfection for immunoblot.

### Cell Viability assay

To detect the proliferation of cells, the Cell Counting Kit-8 (CCK-8) (Dojindo Molecular Technologies, Japan) was used according to the manufacturer’s instructions. In briefly, 3000 cells per well were seeded in 96-well culture plates with Olaparib. Cell viability was determined by WST-8 at a final concentration of 10% to each well, and the absorbance of the samples was measured at 450 nm using a Microplate Reader at 24h as indicated.

### Cell Apoptosis Analysis Using Flow Cytometry

Apoptosis was analyzed by flow cytometry using an Annexin PE-V apoptosis detection kit (Yeasen, Shanghai, China) according to the manufacturer’s instructions. Cells were treated with Olaparib as indicated in the figure legends. Briefly, the cells were washed with cold PBS twice, and then resuspended with 1x Binding buffer and stained with Annexin V/PI reagent in the dark for 15 min. The cells were immediately analyzed by flow cytometry after terminating the staining reaction.

### Statistics

Statistical analyses were performed using GraphPad Prism 8 software. Data of experiments are expressed as mean ± standard deviation (SD) of at least three independent experiments. The Student’s *t* test or one-way analysis of variance was performed to evaluate the differences between two groups or more than two groups. p < 0.05 was considered as statistical significance, and the asterisks represent significance in the following way: *p < 0.05, **p < 0.01, and ***p<0.001.

## Results

### The PARP Inhibitor Olaparib Activates the p53 Pathway

**Figure 1:**
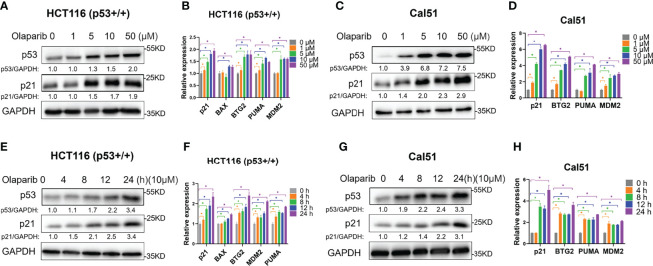
Olaparib treatment activates the expression of p53 and its target genes. **(A, B)** Olaparib treatment elevates the protein **(A)** and mRNA **(B)** levels of p53 and its target genes in a dose-dependent manner in HCT116 ^p53+/+^ cells. **(C, D)** The experiments were performed as the same as **(A, B)** except that Cal51 cells were used. **(E, F)** Olaparib treatment elevates the protein **(E)** and mRNA **(F)** levels of p53 and its target genes in a time-dependent manner in HCT116 ^p53+/+^ cells. Cells were treated with 10 μM of Olaparib and harvested for IB at different time points as indicated. **(G, H)** The experiments were performed as the same as **(E, F)** except that Cal51 cells were used. *p < 0.05.

Our study recently reported that a very low dose of Olaparib induces p53 expression upon RMRP depletion in colorectal cancer cells (29). We are curious to know whether Olaparib regulates p53 expression and activity in the normal culture condition. To test this possibility, we first evaluated the expression of p53 in response to titrated doses of Olaparib, and found that p53 is upregulated in both HCT116 ^p53+/+^ colorectal cancer cells and Cal51 breast cancer cells in a dose-dependent fashion (**Figures 1A, C**). Accordingly, the expression of p53 target genes, including p21, BAX, BTG2, MDM2 and PUMA, was also dose-dependently elevated by Olaparib treatment (**Figures 1A–D**). The minimal dose of Olaparib necessary for competent activation of p53 was about five micromolars in both cell lines as evidenced by the induction of p21 expression (**Figures 1A, C**). In addition, another PARP inhibitor, Niraparib, also induced the expression of p53 and p21 (**Figure S1A**). Next, to further explore the time kinetics of Olaparib-induced p53 activation, we examined p53 and its target gene expression at different time points of 10 μM Olaparib treatment (**Figures 1E-H**). The initial induction of p53 and p21 was first observed at 8 h post-treatment in HCT116 ^p53+/+^ cells and 4 h post-treatment in Cal51 cells (**Figures 1E, G**). Moreover, the expression of multiple p53 target genes were upregulated in HCT116 ^p53+/+^ and Cal51 cell lines in a time-dependent fashion (**Figures 1F, H**). The activation of p53 might not engage other oncogenic signals, as Olaparib could induce p53 in normal ovarian surface epithelial cells, IOSE-80 (**Figure S1B**). Interestingly, we found that Olaparib induces the expression of TIGAR and DRAM1 (**Figures S1C, D**), two p53 target genes critical for glucose metabolism and autophagy, respectively. Therefore, these results indicate that Olaparib treatment leads to activation of the p53 signaling pathway in a dose- and time-dependent manner in colorectal and breast cancer cells.

### Olaparib Induces p53 Stabilization

**Figure 2:**
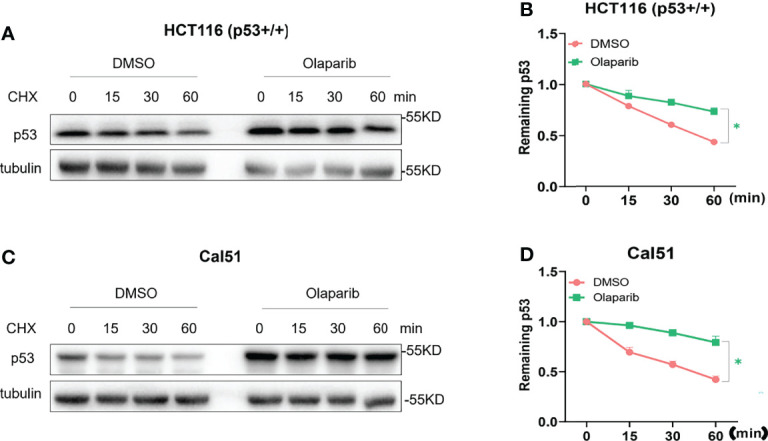
Olaparib treatment promotes p53 stabilization. **(A, B)** HCT116 ^p53+/+^ cells were treated with 10 μM Olaparib for 12 h, and cycloheximide was added to the cultures at different time points before harvest as indicated. Cells were harvested and subject to the IB assay **(A)**, and p53 expression was quantified as shown in the panel **(B)**. **(C, D)** The experiment was performed as the same as **(A, B)** except that Cal51 cells were used. The cycloheximide-chase assay was performed in triplicate, *p < 0.05.

It was shown by our and other groups that activation of p53 is largely due to p53 protein stabilization (18, 29, 34). In keeping with this notion, we also wondered if Olaparib affects p53 protein stability, and therefore performed the cycloheximide-chase analysis of p53 protein half-life. As shown in **Figures 2A, B**, Olaparib treatment indeed led to p53 stabilization as indicated by the prolonged protein half-life in HCT116 ^p53+/+^ cells. Consistently, the p53 half-life was also significantly extended upon Olaparib treatment of Cal51 cells (**Figures 2C, D**). These results reveal that Olaparib can stabilize p53 in different types of cancer cells.

### Olaparib Represses Ribosomal RNA Biogenesis

**Figure 3:**
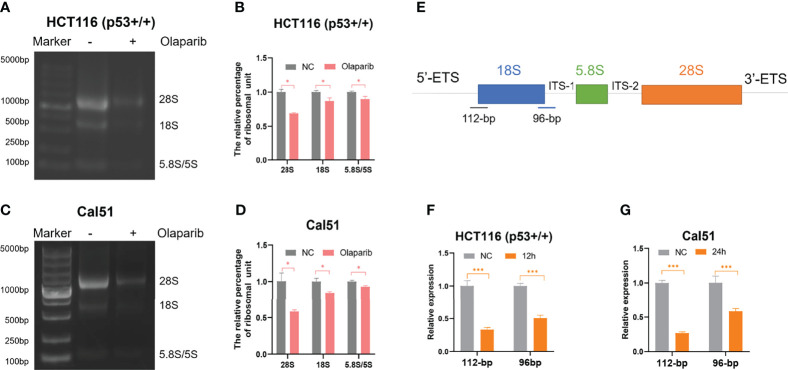
Olaparib treatment inhibits pre-rRNA biosynthesis. **(A, B)** HCT116 ^p53+/+^ cells were treated with or without 10 μM Olaparib for 12 h. The expression of 28S, 18S, and 5.8S rRNA was analyzed by agarose gel electrophoresis **(A)** and quantified as show in the panel **(B)**. **(C, D)** The experiment was performed as the same as **(A, B)** except that Cal51 cells were used. **(E)** A schematic illustration of the pre-rRNAs structure and the fragments amplified by RT-qPCR. **(F, G)** HCT116 ^p53+/+^ **(F)** and Cal51 **(G)** cells were treated with or without 10 μM Olaparib for 24h, and then harvested and subject to RT-qPCR by amplifying a 112-bp fragment through 5’-ETS and 18S rRNA and the other 96-bp fragment encompassing 18S rRNA and ITS-1 as indicated. *p < 0.05 and ***p < 0.001.

Although several studies reported that PARPi may induce p53 activation (26–29), the molecular basis remains unclear. It was previously shown that small nucleolar RNA (snoRNA)-mediated PARP-1 activation contributes to ribosome biogenesis (36). Since we and others have established that impairment of ribosome biogenesis leads to nucleolar stress and consequent p53 activation (31–34), we therefore sought to determine if Olaparib triggers nucleolar stress. We first examined whether Olaparib inhibits biosynthesis of rRNAs, the critical component of the ribosome, by directly comparing 28S, 18S, and 5.8S/5S rRNAs through gel electrophoresis (**Figures 3A–D**). The result showed that Olaparib treatment of both HCT116 ^p53+/+^ (**Figures 3A, B**) and Cal51 (**Figures 3C, D**) cell lines significantly reduces the levels of rRNAs. Importantly, our data also demonstrated that Olaparib suppresses the production of 28S, 18S, and 5.8S/5S rRNAs in a dose- and time-dependent manner in both cell lines (**Figures S2A–D**). p53 was not required for this process, as Olaparib could still inhibit rRNA production in HCT116 ^p53−/−^ cells (**Figure S3**). Biosynthesis of nucleolar rRNAs involves rDNA transcription and pre-rRNA processing into three subtypes of mature rRNAs, including 28S, 18S, and 5.8S rRNAs (32). To elucidate if Olaparib regulates the level of pre-rRNA or not, we performed RT-qPCR by amplifying two fragments encompassing 5’-ETS and 18S rRNA, or 18S rRNA and ITS-1 (35) as indicated in **Figure 3E**. Remarkably, the result clearly showed that Olaparib dramatically reduces the pre-rRNA levels in both cancer cell lines (**Figures 3F, G**). Together, these results suggest that Olaparib treatment can inhibit pre-RNA synthesis to trigger nucleolar stress.

### Olaparib Induces RPL5/RPL11-Dependent p53 Activation

**Figure 4:**
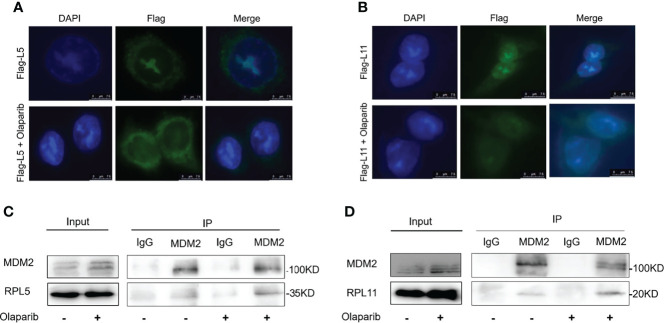
Olaparib treatment enhances the interaction of MDM2 with RPL5 and RPL11. **(A)** HCT116 ^p53+/+^ cells were transfected with Flag-L5 and treated with or without 20 μM Olaparib, and subject to the immunofluorescence assay. **(B)** The experiment was performed as the same as **(A)**, except that Flag-L11 was introduced into cells. **(C)** Cal51 cells were treated with or without 20 μM Olaparib for 24 h and harvested for co-IP-IB analysis. RPL5 was co-immunoprecipitated with MDM2 using an anti-MDM2 antibody. **(D)** The experiment was performed as the same as **(C)**, except that RPL11 was co-immunoprecipitated with MDM2 using an anti-MDM2 antibody.

**Figure 5:**
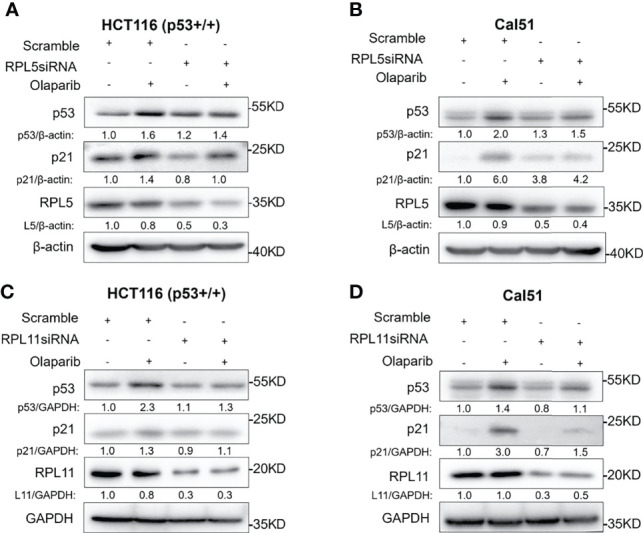
Olaparib-induced p53 activation requires RPL5 and RPL11. **(A, B)** HCT116 ^p53+/+^ **(A)** and Cal51 **(B)** cells were transfected with control or RPL5 siRNA for 24 h, and then treated with or without 10 μM Olaparib for another 24 h before harvest for IB analysis. **(C, D)** The experiments were performed as the same as **(A, B)** except that RPL11 siRNA was used.

Since nucleolar stress elicits the release of RPL5 and RPL11 into the nucleus where they repress MDM2-induced p53 degradation by directly binding to MDM2 (31–34, 37), we attempted to elaborate if Olaparib also provokes the RPs-MDM2-p53 cascade by inducing nucleolar stress. First, we conducted the immunofluorescence assay and found that Flag-L5 and Flag-L11 are mainly localized in the nucleolus (for pre-ribosome assembly) and the cytoplasm (for protein translation) in untreated cells, while the nucleolar localization of RPL5 and RPL11 are disrupted in response to Olaparib treatment (**Figures 4A, B**). Next, by performing a set of co-IP assays using the anti-MDM2 antibody, we showed that Olaparib treatment indeed enhances the interaction between MDM2 and both RPs, respectively (**Figures 4C, D**). Furthermore, we wondered if RPL5 and RPL11 are required for p53 activation in response to Olaparib-induced nucleolar stress. As shown in **Figure 5**, depletion of RPL5 with siRNA markedly inhibited Olaparib activation of p53 in HCT116 ^p53+/+^ and Cal51 cells (**Figures 5A, B**). Consistently, the same effect was achieved by knocking down RPL11 in both cell lines (**Figures 5C, D**). Taken together, these results demonstrate that Olaparib activation of p53 requires RPL5 and RPL11 binding to MDM2.

### Olaparib Suppresses Cancer Cell Growth Partially Dependent on p53

**Figure 6:**
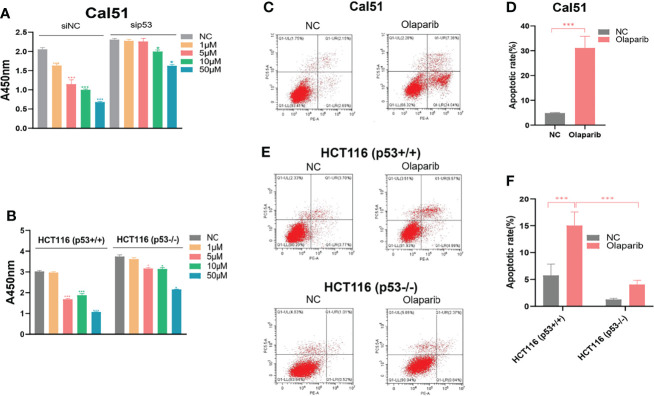
Olaparib suppresses growth and prompts apoptosis of cancer cells. **(A, B)** Cell viability assay was performed to assess the growth of Cal51, p53-depleted Cal51, HCT116 ^p53+/+^ and HCT116 ^p53−/−^ cells upon Olaparib treatment as indicated. **(C, D)** Apoptosis of Cal51 cells treated with or without Olaparib was assessed by flow cytometry. **(E, F)** Apoptosis of HCT116 ^p53+/+^ and HCT116 ^p53−/−^ cells treated with or without Olaparib was assessed by flow cytometry. *p < 0.05 and ***p<0.001.

Given the action of Olaparib to trigger the nucleolar stress-p53 pathway, we determined if the p53 status is correlated with cytotoxic effect of Olaparib by using the wild-type p53-harboring HCT116 ^p53+/+^ and Cal51 cell lines and the p53-null HCT116 ^p53−/−^ cell line. The cell viability assay was performed to show that Olaparib significantly represses HCT116 ^p53+/+^ and Cal51 cell growth in a dose-dependent manner (**Figures 6A, B**), which is in line with the former results (**Figures 1A–D**). It was noted that as low as 5 μM Olaparib is able to markedly suppress Cal51 and HCT116 ^p53+/+^ cell proliferation (**Figures 6A, B**), probably because p53 can be fully activated at this dosage (**Figures 1A, C**). More importantly, the p53-depleted Cal51 and HCT116 ^p53−/−^ cell lines exhibited much lower sensitivity to Olaparib compared to their isogenic counterparts (**Figures 6A, B**). Moreover, we also examined the effect of Olaparib on apoptosis in these cell lines. Consistently, Olaparib drastically induced apoptosis of HCT116 ^p53+/+^ and Cal51 cells, while had a moderate effect on the induction of apoptosis in HCT116 ^p53−/−^ cells (**Figures 6C–F**). Finally, we showed that Olaparib triggers cell cycle arrest at G2 phase in HCT116 ^p53+/+^ (**Figures S4A, B**) and Cal51 cells (**Figures S4C, D**), which is in line with the former results that Olaparib induces p21 expression (**Figure 1**). Therefore, our results demonstrate that Olaparib inhibits cell proliferation and promotes apoptosis partially dependent on p53.

## Discussion

**Figure 7:**
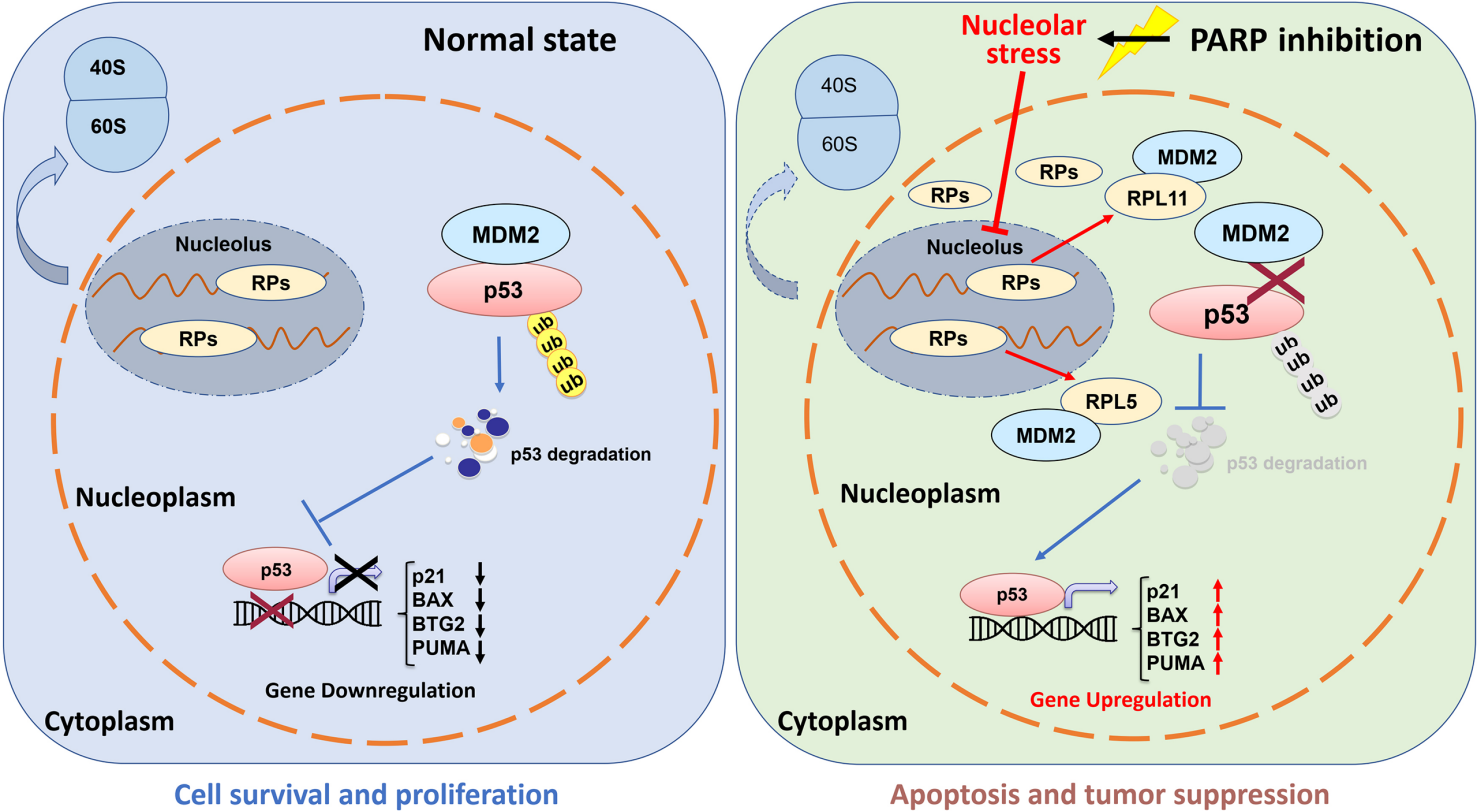
Working model of Olaparib activation of p53 *via* nucleolar stress. Under the normal condition, RPs and rRNAs work together for pre-ribosome assembly in the nucleolus, while MDM2 binds to p53 and maintains a relatively low level of p53 in cells (left panel). Olaparib treatment inhibits pre-rRNA biosynthesis, thus leading to nucleolar stress. Many RPs, such as RPL5 and RPL11, are released to the nucleoplasm to interact with MDM2 resulting in p53 stabilization and activation, and consequent p53 target gene upregulation.

The tumor suppressor p53 plays a vital role in preventing tumorigenesis by regulating the expression of a myriad of genes involved in DNA damage response and apoptosis. Inactivation of p53 usually leads to cancer development and therapeutic resistance (18, 19, 29, 38, 39). In this study, we showed that Olaparib treatment promotes p53 protein stabilization and thus upregulates p53 target gene expression in a dose- and time-dependent manner (**Figures 1**, **2**). Mechanistically, Olaparib was found to trigger nucleolar stress by inhibiting pre-rRNA biosynthesis (**Figure 3**), consequently leading to enhanced interaction between RPL5/RPL11 and MDM2 (**Figure 4**). Conversely, knockdown of RPL5 or RPL11 by siRNAs markedly impaired Olaparib-induced p53 activation (**Figure 5**). More importantly, Olaparib suppressed breast and colorectal cancer cell survival and proliferation partially through activation of p53 (**Figure 6**). Taken together, our study uncovers an unexplored therapeutic action of PARPi by activating the p53 pathway. MDM2 binds to and promotes degradation of p53 under the normal condition, while PARPi induces nucleolar stress to release RPL5 and RPL11 into the nucleoplasm, enhancing the interactions between the RPs and MDM2 and, consequently leading to p53 stabilization and activation (**Figure 7**).

PARPi has been widely used in the treatment of tumors with *BRCA1/2* mutation or HRD (13, 14). The *TP53* gene status was also reported to be associated with tumor sensitivity to PARPi. Several studies suggest that *TP53* mutation predicts enhanced cytotoxicity of PARPi, because inactivation of p53 accelerates cell cycle progression and impairs the DDR pathways, thus accumulating unrepaired DNA damage (40). Alternatively, missense mutant p53 can associate with PARP-1 to promote aberrant repair of the damaged DNA caused by the alkylating agent, which may create a strong tumor dependency on PARP-1 (41, 42). By contrast, PARPi was also shown to repress wild-type p53-harbroing tumors by inducing p53-dependent apoptosis or ferroptosis (26, 27, 43). In addition, we have recently reported that depletion of RMRP elicits full activation of p53 under Olaparib treatment, leading to tumor sensitization to PARPi-associated therapies (29). These seemingly contradictory findings indicate that p53-dependent DDR and cell death play distinct roles in PARPi treatment of cancer. However, it is not very clear how PARPi activates p53. We also showed that Olaparib-induced p53 activation is coincident with the elevation of phosphorylation of γ-H2AX, a maker for DNA damage (**Figures S5A, B**). One possible mechanism is that PARPi-caused replication stress and the consequent DNA damage stress may induce p53 activation, though the detailed mechanism is yet to be investigated. In this study, nevertheless, we clearly demonstrate that PARPi activation of p53 involves perturbation of ribosome biogenesis and interaction of the ribosome-free RPs with MDM2, which provides the first mechanistic insight into how these agents activate the p53 pathway. Remarkably, our study suggests that PARP and the nucleolus may be the dual targets for PARPi, and that these agents could be used in tumors with HRD and/or active ribosome biogenesis.

It has long been noticed that the nucleoli are morphologically altered in transformed or cancer cells (44, 45), because these cells often sustain a high rate of ribosome biogenesis to fulfill the requirement for their own rapid growth and propagation. Thus, interference with rRNA and RP synthesis or ribosome assembly, which causes nucleolar stress and consequent p53 activation, has become a promising anti-cancer strategy (31, 32). It was found that a low dose of Actinomycin D (<10 nM) selectively inhibits rDNA transcription, although it may also lead to DNA damage stress at a higher dose. In addition, several DNA damage-based therapies, such as 5-Fluorouracil, Cisplatin, Doxorubicin, and UV or γ-irradiation, are able to induce nucleolar stress by repressing rDNA transcription or rRNA processing. Some RPs, such as RPL37, were reported to undergo degradation in response to the genotoxic insults, Cisplatin and UV light. Moreover, mycophenolic acid, an immunosuppressant drug, was found to disturb the nucleolar architecture and impair rRNA synthesis. Recently, several small molecules with anti-cancer activity have been developed to selectively inhibit rRNA production. CX-3543 was identified as an inhibitor of G-quadruplexes that are crucial to transcription of GC-rich rDNAs (46). Another nucleolar stress-inducing agent CX-5461 impedes recruitment of SL1, a critical component of the RNA Pol I initiation complex, on the rDNA promoter (47). BMH-21 associates with GC-rich rDNA genes to suppress RNA Pol I function, as well as promotes proteasomal degradation of the RNA Pol I catalytic subunit RPA194 (48). In our attempt to elucidate the molecular basis behind PARPi activation of p53, we showed that Olaparib treatment markedly inhibits production of pre-rRNAs, leading to reduced levels of the 28S, 18S, and 5.8S rRNAs (**Figure 3**), which is in line with a previous study showing that PARylation of the RNA helicase DDX21 by PARP-1 facilitates rDNA transcription (36). As expected, we further demonstrated that the inhibition of rRNA biosynthesis leads to nucleolar stress in which ribosome free-RPL5 and -RPL11 interact with MDM2 to stabilize p53 (**Figures 4**, **5**). Nevertheless, a few questions are also raised based on our findings. Whether or not other RPs and ribosome-related proteins, such as RPL23 (49, 50), RPS14 (33), and SBDS (34), are involved in PARPi-induced p53 activation remains to be understood. Since the *TP53* gene is mutated in around 50% of human cancers, it is worthwhile to investigate if PARPi-triggered nucleolar stress regulates mutant p53 signaling. Given that ribosome-free RPs also interact with TAp73 (51) and c-Myc (52), it is intriguing to test if PARPi modulates these signaling pathways independently of p53 *via* eliciting nucleolar stress.

## Conclusion

PARPi have been widely used for treatment of tumors harboring *BRCA1/2* mutation or with HRD as a synthetic lethal agent. Recent studies revealed that PARPi can induce p53-dependent cell death that contributes to the anti-cancer effect of this agent. However, the molecular mechanism underlying how PARPi activates the p53 pathway is elusive. In this study, we demonstrate for the first time that Olaparib suppresses rRNA biosynthesis, thus eliciting the nucleolar stress-RPs-p53 axis and consequent cancer cell apoptosis. Our study also suggests that rRNA biogenesis could be an alternative target for PARPi, which is worthwhile for clinical test in future.

## Data Availability Statement

The original contributions presented in the study are included in the article/supplementary material. Further rational inquiries can be directed to the corresponding authors.

## Ethics Statement

The study was approved by both the Ethics Committee of Xinxiang Medical University and Fudan University Shanghai Cancer Center.

## Author Contributions

TH conducted and analyzed part of the experiments and provided critical reagents and materials. JT conducted and analyzed most of the experiments. MW and YG performed part of IB analysis. BG, JC, and YL provided important instructions and helped to analyze the data. TH, QH and XZ conceived, designed and supervised the study, and analyzed the data. TH, JT and XZ drafted the manuscript. QH and XZ edited the manuscript. All authors contributed to the article and approved the submitted version. All authors contributed to the article and approved the submitted version.

## Funding

TH was supported by the National Natural Science Foundation of China (Nos. 82002731 and 82172891), the Henan Natural Science Fund for Excellent Young Scholars (No. 212300410067) and Doctoral Foundation of Xinxiang Medical University (No. XYBSKYZZ202001). XZ was supported by the National Natural Science Foundation of China (Nos. 81874053 and 82072879), and QH was supported by the National Natural Science Foundation of China (No. 82173022).

## Conflict of Interest

The authors declare that the research was conducted in the absence of any commercial or financial relationships that could be construed as a potential conflict of interest.

## Publisher’s Note

All claims expressed in this article are solely those of the authors and do not necessarily represent those of their affiliated organizations, or those of the publisher, the editors and the reviewers. Any product that may be evaluated in this article, or claim that may be made by its manufacturer, is not guaranteed or endorsed by the publisher.
