# Supplementary material for: Olaparib Induces RPL5/RPL11-Dependent p53 Activation via Nucleolar Stress
Source: Front Oncol. 2022 Jun 3;12:821366. doi: 10.3389/fonc.2022.821366 (PMC9204002; doi:10.3389/fonc.2022.821366)
Supplement: Supplementary file 1 [file DataSheet_1.pdf]

Olaparib induces RPL5/RPL11-dependent p53 activation via nucleolar stress

Tao Han[1,#], Jing Tong[2,3,#], Mengxin Wang[1], Yu Gan[4], Bo Gao[2], Jiaxiang Chen[4], Youxun Liu[1], Qian Hao[2,3,*], and Xiang Zhou[2,3,5,6*]

[1] School of Basic Medical Sciences, Xinxiang Medical University, Xinxiang 453003, China
[2] Fudan University Shanghai Cancer Center, Fudan University, Shanghai 200032, China
[3] Department of Oncology, Shanghai Medical College, Fudan University, Shanghai 200032, China
[4] Department of Physiology, Medical College of Nanchang University, Nanchang, 330006, China
[5] Key Laboratory of Breast Cancer in Shanghai, Fudan University Shanghai Cancer Center, Fudan University, Shanghai, 200032, China
[6] Shanghai Key Laboratory of Medical Epigenetics, International Co-laboratory of Medical Epigenetics and Metabolism (Ministry of Science and Technology), Institutes of Biomedical Sciences, Fudan University, Shanghai 200032, China

Supplementary Figure Legends

Figure S1. (A) Niraparib treatment induces the expression of p53 and p21 in Cal51 cells. (B) Olaparib treatment activates p53 in normal ovarian surface epithelial cells, IOSE-80. (C) Olaparib treatment induces the expression of TIGAR and DRAM1 in Cal51 cells. (D) Olaparib treatment induces the expression of TIGAR and DRAM1 in HCT116 $^{p53+/+}$ cells.

Figure S2. (A) Olaparib treatment inhibits rRNA production in a dose-dependent manner in HCT116 $^{p53+/+}$ cells. (B) Olaparib treatment inhibits rRNA production in a dose-dependent manner in Cal51 cells. (C) Olaparib treatment inhibits rRNA production in a time-dependent manner in HCT116 $^{p53+/+}$ cells. (D) Olaparib treatment inhibits rRNA production in a time-dependent manner in Cal51 cells.

Figure S3. Olaparib treatment inhibits rRNA production in HCT116 $^{p53-/-}$ cells.

Figure S4. (A, B) Olaparib treatment triggers G2 phase accumulation in HCT116 $^{p53+/+}$ cells. (C, D) Olaparib treatment triggers G2 phase accumulation in Cal51 cells.

Figure S5. (A) Olaparib treatment increases phosphorylation of γ-H2AX in HCT116 $^{p53+/+}$ cells. (B) Olaparib treatment increases phosphorylation of γ-H2AX in Cal51 cells.

Figure S1

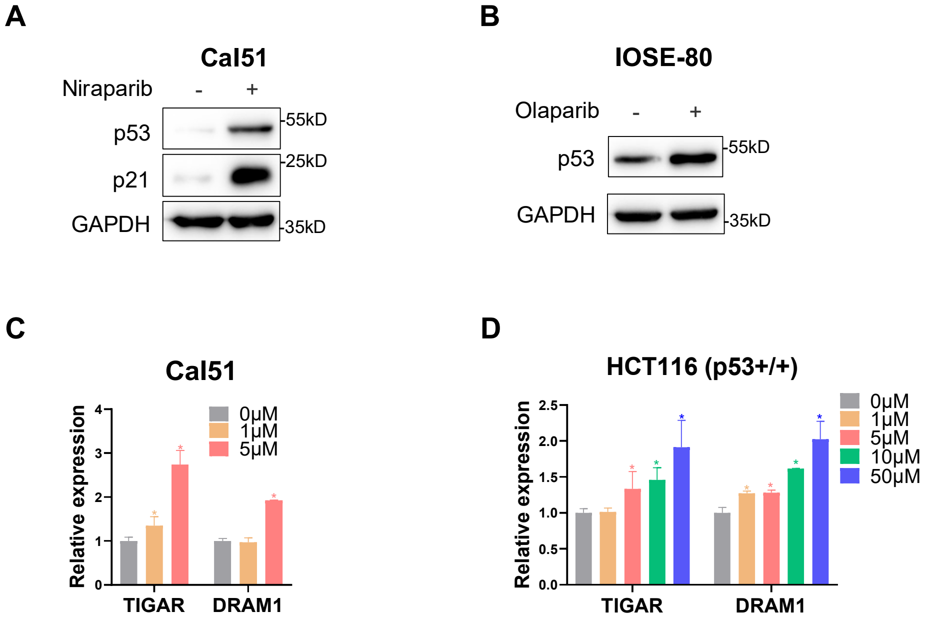
A
Cal51
Niraparib - +
p53
p21
GAPDH
55kD
25kD
35kD
B
IOSE-80
Olaparib - +
p53
GAPDH
55kD
35kD
C
Cal51
Relative expression
0µM
1µM
5µM
TIGAR
DRAM1
D
HCT116 (p53+/+)
Relative expression
0µM
1µM
5µM
10µM
50µM
TIGAR
DRAM1

Figure S2

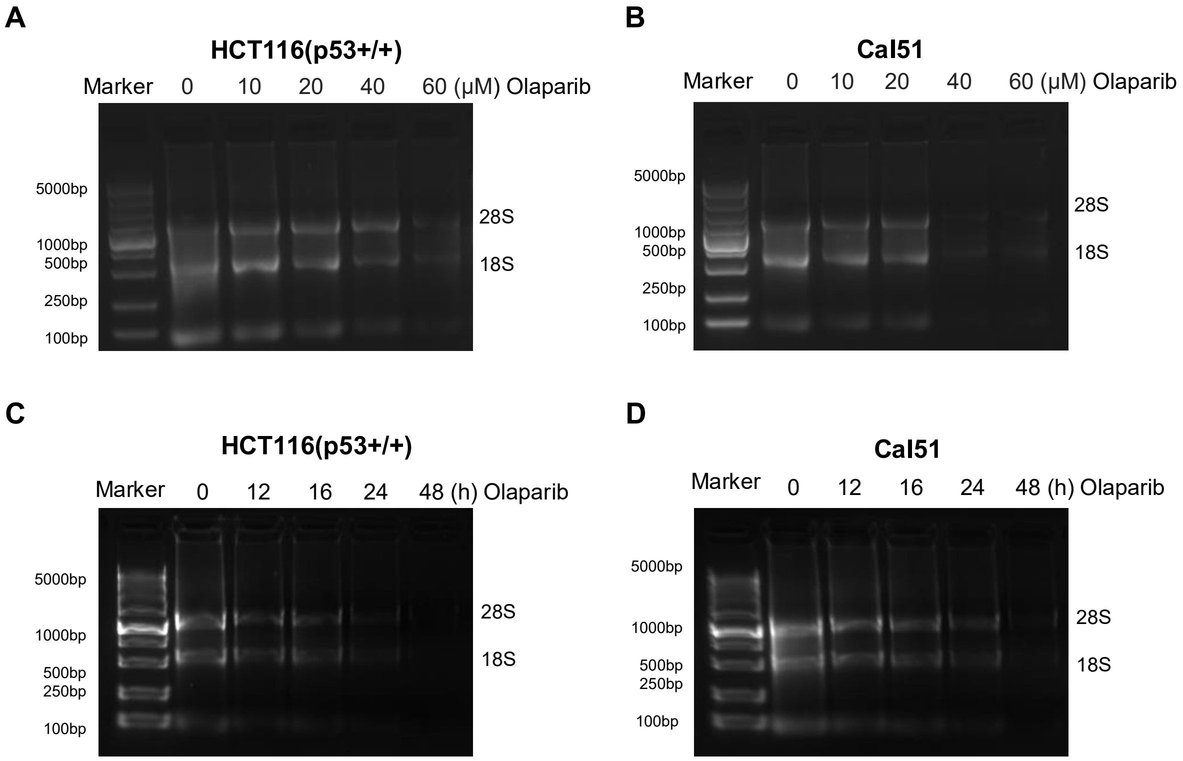
A
HCT116(p53+/+)
Marker 0 10 20 40 60 (μM) Olaparib
5000bp
1000bp
500bp
250bp
100bp
28S
18S
B
Cal51
Marker 0 10 20 40 60 (μM) Olaparib
5000bp
1000bp
500bp
250bp
100bp
28S
18S
C
HCT116(p53+/+)
Marker 0 12 16 24 48 (h) Olaparib
5000bp
1000bp
500bp
250bp
100bp
28S
18S
D
Cal51
Marker 0 12 16 24 48 (h) Olaparib
5000bp
1000bp
500bp
250bp
100bp
28S
18S

Figure S3

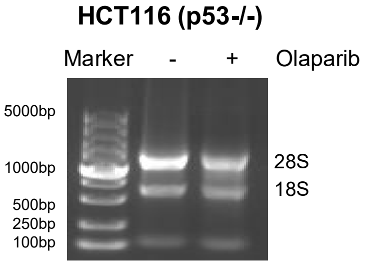
HCT116 (p53-/-)
Marker
-
+
Olaparib
5000bp
1000bp
500bp
250bp
100bp
28S
18S

Figure S4

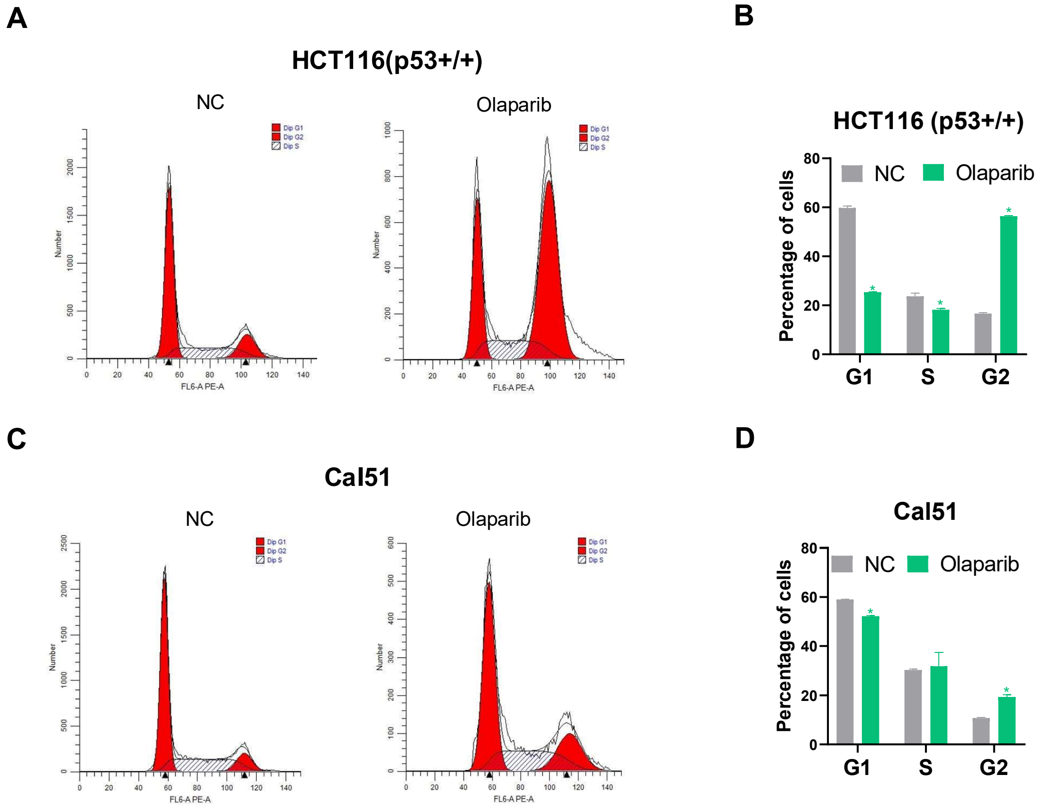
A
HCT116(p53+/+)
NC
Olaparib
Dip G1
Dip G2
Dip S
Number
FL6-A PE-A
B
HCT116 (p53+/+)
NC
Olaparib
Percentage of cells
G1
S
G2
C
Cal51
NC
Olaparib
Dip G1
Dip G2
Dip S
Number
FL6-A PE-A
D
Cal51
NC
Olaparib
Percentage of cells
G1
S
G2

Figure S5

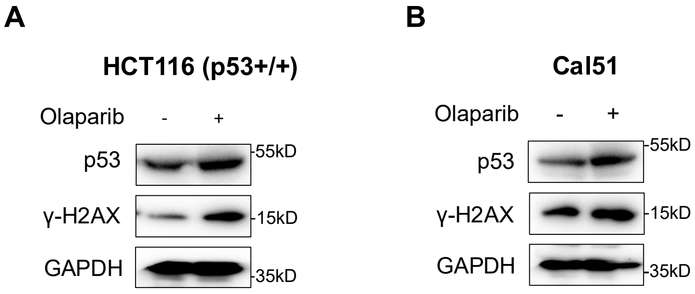
A
HCT116 (p53+/+)
Olaparib
-
+
p53
55kD
γ-H2AX
15kD
GAPDH
35kD
B
Cal51
Olaparib
-
+
p53
55kD
γ-H2AX
15kD
GAPDH
35kD
